# Bone metastases identify a high-risk subgroup in metastatic neuroendocrine tumors with particular relevance in NETs of unknown primary

**DOI:** 10.3389/fendo.2026.1906789

**Published:** 2026-10-28

**Authors:** Annie Mathew, Anna Kyewski, Pia Hauptmeier, David Kersting, Benedikt M. Schaarschmidt, Dagmar Fuhrer, Harald Lahner

**Affiliations:** 1 Department of Endocrinology, Diabetes and Metabolism and Division of Laboratory Research, University Hospital Essen, Endocrine Tumor Center at WTZ/Comprehensive Cancer Center and ENETS Center of Excellence, University of Duisburg-Essen, Essen, Germany; 2 Center of Endocrine Medicine, University of Duisburg-Essen, Essen, Germany; 3 Department of Nuclear Medicine, West German Cancer Center, German Cancer Consortium (DKTK) and National Center for Tumor Diseases site, University Hospital Essen, University of Duisburg-Essen, Essen, Germany; 4 Institute for Diagnostic and Interventional Radiology and Neuroradiology, University Hospital Essen, University of Duisburg-Essen, Essen, Germany

**Keywords:** bone metastases, cancer of unknown primary, neuroendocrine tumors (NET), overall survival, pancreatic neuroendocrine tumor (PanNET), small intestinal neuroendocrine tumors, somatostatin receptor imaging

## Abstract

**Background:**

Bone metastases (BM) are an increasingly recognized manifestation of advanced neuroendocrine tumors (NETs), but their prevalence and association with survival in NETs of unknown primary (CUP NET) remain insufficiently characterized. We assessed the prevalence of BM and their association with overall survival (OS) in patients with metastatic pancreatic NETs (PanNETs), small intestinal NETs (siNETs), and CUP NETs.

**Methods:**

We conducted a retrospective analysis of 642 patients with histologically confirmed metastatic well-differentiated NETs who were treated at the European Neuroendocrine Tumour Society (ENETS) Center Essen between 2009 and 2023 using somatostatin-receptor (SSTR)- based hybrid imaging with 68Ga-DOTATOC PET/CT. The patients included 171 with PanNETs, 305 with siNETs, and 166 with CUP NETs. The primary outcome was OS, defined as the time from date of initial NET diagnosis to death from any cause. Secondary outcomes included BM prevalence, entity-specific survival according to BM status, BM morphology and timing, and skeletal-related events.

**Results:**

BM were detected in 238 patients (37.1%) and were most frequent in CUP NETs (42.8%), followed by PanNETs (36.3%) and siNETs (34.4%). Median OS in the overall cohort was 77 months (95% confidence interval [CI], 64–89) in patients with BM and 141 months (95% CI, 119–186) in metastatic patients without BM (log-rank p < 0.001). In CUP NETs, median OS was 30 months in patients with BM and 66 months in metastatic patients without BM (log-rank p = 0.021). In multivariable Cox regression adjusted for age, sex, tumor grade, functioning status, and tumor entity, BM remained associated with shorter OS (hazard ratio [HR], 1.45; 95% CI, 1.13–1.85; p = 0.0034). In an entity-stratified model BM remained associated with OS (HR, 1.43; 95% CI, 1.09–1.89; p = 0.011). In the CUP NET subgroup, the adjusted HR associated with BM was 1.83 (95% CI, 1.17–2.85; p = 0.008).

**Conclusion:**

BM were common in patients with metastatic NETs and are particularly prevalent and clinically relevant in CUP NET.

## Introduction

Neuroendocrine tumors (NETs) are a heterogeneous group of neoplasms characterized by diverse biological behavior and clinical outcomes. In advanced disease, distant metastases are a major determinant of prognosis. However, skeletal involvement is increasingly recognized as a clinically relevant manifestation in oncological patients, associated with pain, skeletal-related events (SRE), impaired quality of life and reduced survival (1).

Bone metastases (BM) are described as a negative prognostic factor in several solid malignancies. In NETs, recent studies indicate that BM occur more frequently than previously assumed, particularly in advanced pancreatic (PanNET) and small intestinal NET (siNET) (2, 3). BM were associated with unfavorable outcomes in these entities, suggesting that skeletal dissemination reflects aggressive tumor biology rather than a late incidental finding. Nevertheless, the reported prevalence of BM in NETs varies widely, depending on tumor entity, disease stage and imaging modalities used for detection.

Neuroendocrine tumors of unknown primary (CUP NET) represent a distinct and poorly characterized subgroup within the NET spectrum (4). By definition, CUP NETs lack an identifiable primary tumor despite thorough diagnostic evaluation and often present with advanced metastatic disease. Occult small intestinal, pancreatic, bronchopulmonary, or other primary tumors may remain undetected, and the diagnostic workup can vary between patients and over time. Consequently, detailed characterization of the diagnostic procedures used to establish CUP NET is essential when interpreting clinical outcomes in this population (5).

Data on the frequency, characteristics, and prognostic relevance of BM in CUP NETs remain limited. We therefore analyzed a retrospective cohort of patients with metastatic, well-differentiated PanNETs, siNETs, and CUP NETs treated at a tertiary institution (European Neuroendocrine Tumor Society (ENETS) Center of Excellence, Essen). The primary objective was to evaluate the association between BM and OS. Secondary objectives were to describe the prevalence, timing, morphology, and clinical complications of BM across NET entities and to examine the association between BM and OS specifically within the CUP NET subgroup.

## Materials and Methods

### Study design and patient population

We conducted a retrospective, single-center cohort study using data from a prospectively maintained NET database at the ENETS Center of Excellence, Department of Endocrinology, Diabetes and Metabolism, University Hospital Essen. Patients treated between January 2009 and December 2023 were screened for eligibility.

Eligible patients were required to have:

1. histologically confirmed neuroendocrine differentiation;
2. a well-differentiated NET classified as grade 1 (G1), grade 2 (G2), or well-differentiated grade 3 (G3);
3. metastatic disease;
4. a pancreatic, small intestinal, or unknown primary tumor site; and
5. available clinical follow-up and survival information.
6. Patients with poorly differentiated neuroendocrine carcinoma or mixed neuroendocrine– non-neuroendocrine neoplasms were excluded.

All cases were discussed by a multidisciplinary NET tumor board. The study was approved by the Ethics Committee of the Medical Faculty of the University of Duisburg-Essen (18-8367-BO).

### Definition and diagnostic evaluation of CUP NET

CUP NET was defined as a histologically confirmed metastatic, well-differentiated NET for which no primary tumor site could be identified after completion of the diagnostic evaluation available at the time of presentation.

The routine diagnostic workup comprised review of the clinical history, physical examination, laboratory testing, and 68Ga-DOTATOC PET/CT with diagnostic contrast-enhanced CT of the chest, abdomen, and pelvis (68Ga-DOTATOC PET/CT). Additional investigations were performed according to clinical presentation and imaging findings and included: gastroscopy, colonoscopy with terminal ileoscopy, endoscopic ultrasound, or bronchoscopy. Diagnostic evaluation was based on recommendations available during the study period, including ENETS guidance (4, 6).

Occult small intestinal primaries were assessed using 68Ga-DOTATOC PET/CT, multiphasic CT/MRI, surgical exploration, or combinations thereof. Because not all patients underwent the same invasive or small-bowel-specific investigations, a clinically occult small intestinal primary cannot be definitively excluded in every CUP NET case. This limitation was considered when interpreting entity-specific findings.

### Pathological assessment

Histopathological diagnosis was established from the primary tumor or a metastatic lesion. Tumors were classified as well-differentiated NETs according to the World Health Organization criteria applicable at the time of diagnosis. Tumor grade was determined using the Ki-67 proliferation index. Ki-67 values were available in 626/642 (97.5%) of the overall cohort and in 165/166 (99.4%) of CUP NET cases. A valid Ki-67 index measurement could not be obtained for a total of 16 patients due to fragmented tumor samples. All of these patients were histopathologically diagnosed with a differentiated neuroendocrine neoplasm.

For CUP NETs, immunohistochemical markers potentially suggestive of the primary site, including CDX2, cytokeratin 7 (CK7), cytokeratin 20 (CK20), special AT-rich sequence-binding protein 2 (SATB2), paired box gene 8 (PAX8), insulin gene enhancer protein ISL1, and thyroid transcription factor 1 (TTF1), were recorded. CDX2, ISL1, and TTF1 were tested routinely in all cases of CUP NET; the other markers were tested as clinically indicated. Immunohistochemical profiles were used as supportive information but were not considered definitive evidence of a primary site in the absence of an anatomically identifiable lesion.

### Identification and characterization of bone metastases

BMs were diagnosed using repeated 68Ga-DOTATOC PET/CT scans with diagnostic contrast-enhanced CT of the chest, abdomen, and pelvis, which were incorporated into the routine staging process for all patients. A lesion was classified as a BM when imaging demonstrated findings considered compatible with osseous metastatic disease and the diagnosis was confirmed by multidisciplinary review. In the SSTR-directed imaging part, focal osseous tracer uptake exceeding the surrounding physiological bone background and not attributable to a benign process was considered suspicious for BM. On CT, osteoblastic, osteolytic, or mixed lesions with a distribution and appearance compatible with metastatic disease were included. Equivocal findings were resolved by correlation with previous or follow-up imaging, an additional imaging modality, or histopathology when available.

BM were classified as present at initial diagnosis or detected during follow-up. “Initial BM” was defined as BM documented within six months of the date of initial NET diagnosis; BM detected thereafter were classified as metachronous or acquired BM.

### Outcomes

The primary outcome was OS, defined as the interval from initial NET diagnosis to death from any cause. Patients alive at the last documented clinical contact were censored on that date.

Secondary outcomes were:

- prevalence of BM in the entire cohort and by NET entity;
- entity-specific OS according to BM status;
- association between BM and OS within the CUP NET subgroup;
- timing and radiological morphology of BM;
- frequency and type of skeletal-related events (SREs); and
- use of antiresorptive therapy.

SREs were defined as pathological fracture, spinal cord compression, radiotherapy or surgery to bone, or clinically significant bone pain requiring intervention.

### Covariates

The following variables were extracted: age at diagnosis, sex, NET entity, Ki-67 proliferation index, tumor grade, and functioning status. A functioning tumor was defined as a NET associated with a clinically documented hormone-related syndrome rather than biochemical hormone secretion alone. Variables for multivariable modeling were selected *a priori* on the basis of established prognostic relevance in metastatic NETs and their plausible association with BM. The model included BM status, age, sex, tumor grade, functioning status, and tumor entity.

### Statistical analysis

Continuous variables are presented as medians with interquartile ranges (IQRs), and categorical variables as counts and percentages. Survival distributions were estimated using the Kaplan–Meier method and compared using two-sided log-rank tests. Univariable and multivariable Cox proportional hazards regression models were used to estimate associations with OS. Results are reported as hazard ratios (HRs) with 95% confidence intervals (CIs).

The proportional hazards assumption was violated for tumor entity. Consequently, an additional Cox proportional hazards model was fitted with stratification by NET entity, allowing the baseline hazard to vary across PanNET, siNET, and CUP NET, while estimating a common hazard ratio for the remaining covariates.

All tests were two-sided, and p values below 0.05 were considered statistically significant. Statistical analyses were performed using R 4.5.2 (R Foundation for Statistical Computing, Vienna, Austria).

## Results

### Patient characteristics

A total of 642 patients with metastatic, well-differentiated NETs were included, comprising 171 patients with PanNETs, 305 with siNETs, and 166 with CUP NETs. All patients had stage IV disease. The median follow-up was 50 months (IQR, 25–92 months). Baseline demographic and tumor characteristics are summarized in **Table 1**. Median age at diagnosis differed across NET entities (p = 0.001): 56.0 years (IQR, 47.0–65.0) in PanNETs, 59.0 years (IQR, 50.0–67.0) in siNETs, and 61.0 years (IQR, 51.2–71.8) in CUP NETs.

**Table 1:** Baseline characteristics of the study cohort by neuroendocrine tumor entity. Data are presented as median [IQR] or n (%).

Variable	PanNET (n = 171)	siNET (n = 305)	CUP NET (n = 166)	p value
Age at diagnosis, years	56.0 [47.0-65.0]	59.0 [50.0-67.0]	61.0 [51.2-71.8]	0.001
Male sex, n (%)	86 (50.3)	173 (56.7)	88 (53.0)	0.33
Tumor grading, n (%)				<0.001
G1	36 (21.7)	174 (57.1)	57 (34.5)	
G2	106 (63.9)	121 (39.7)	82 (49.7)	
G3	24 (14.5)	8 (2.6)	26 (15.8)	
Functioning tumor, n (%)	20 (11.7)	171 (56.1)	73 (44.0)	<0.001
BM present, n (%)	62 (36.3)	105 (34.4)	71 (42.8)	0.14
Median OS, months	82 (71–121)	170 (144–230)	48 (30–64)	<0.001

PanNET, pancreatic NET; siNET, small-intestinal NET, CUP-NET, neuroendocrine tumor of unknown primary; BM, bone metastases; OS, overall survival; IQR, interquartile range.

Tumor-grade distributions also differed between entities (p < 0.001). G1 tumors accounted for 21.7% of PanNETs, 57.1% of siNETs, and 34.5% of CUP NETs. G2 tumors accounted for 63.9%, 39.7%, and 49.7%, respectively, and well-differentiated G3 tumors for 14.5%, 2.6%, and 15.8%, respectively. Tumor grade was unavailable for eight patients of the entire cohort (1.3%; 5/171 PanNET, 2/305 siNET, 1/166 CUP NET). Functioning tumors were documented in 20 patients with PanNETs (11.7%), 171 with siNETs (56.1%), and 73 with CUP NETs (44.0%; p < 0.001). BM were identified in 238 patients (37.1%). Their prevalence was 36.3% in PanNETs, 34.4% in siNETs and 42.8% in CUP NETs. The difference between entities was not statistically significant (p = 0.14).

### Overall survival

In the overall cohort, median OS was 77 months (95% CI, 64–89) among patients with BM and 141 months (95% CI, 119–186) among patients without BM (log-rank p < 0.001; **Figure 1**).

**Figure 1:**
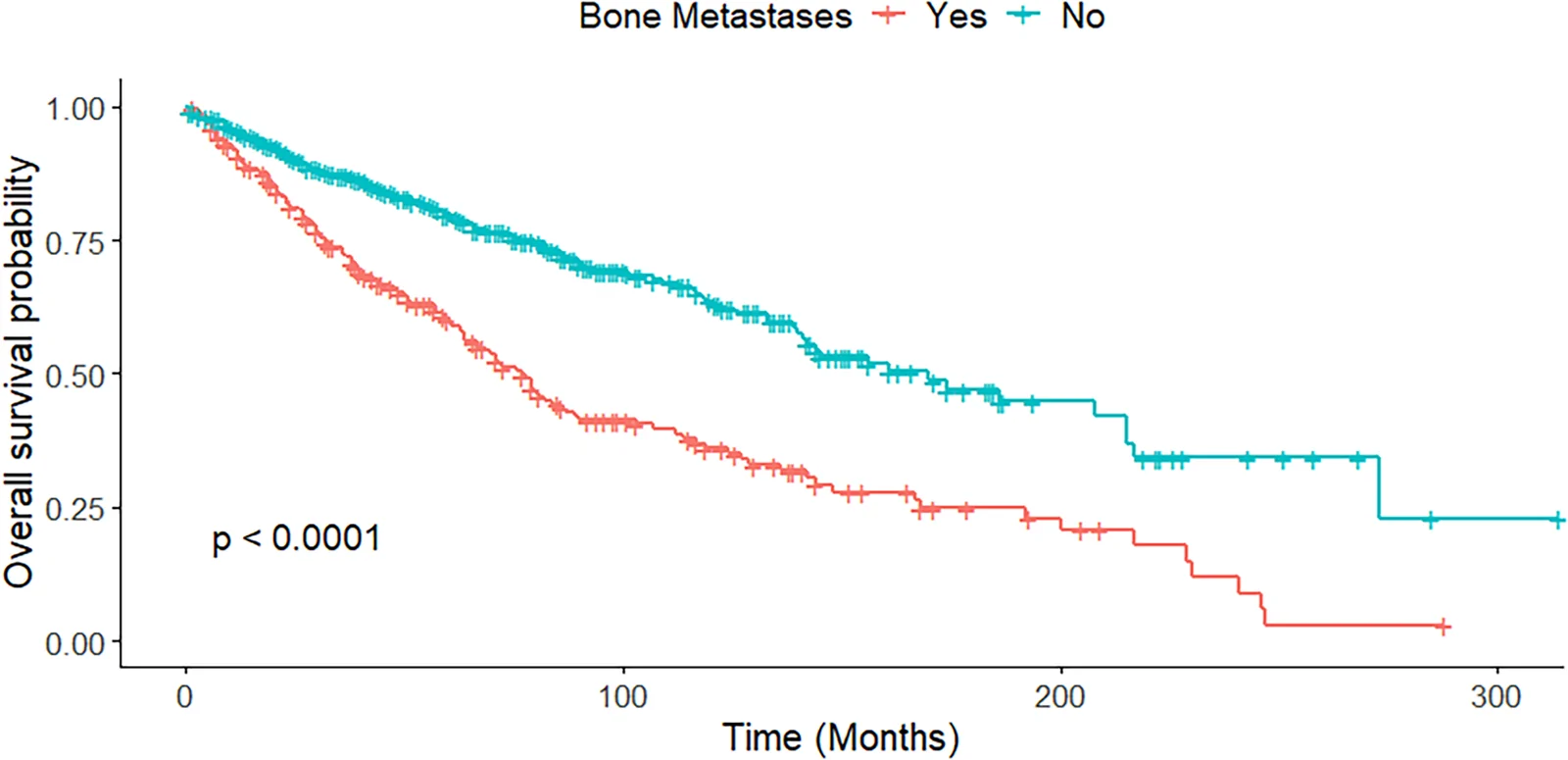
Kaplan-Meier estimates of overall survival according to bone metastases status in patients with neuroendocrine tumors.

Entity-specific Kaplan–Meier analyses are shown in **Figure 2**. In CUP NETs, median OS was 30 months in patients with BM and 66 months in patients without BM (log-rank p = 0.021). In PanNETs, median OS was 63 months and 116 months, respectively (log-rank p = 0.016). In siNETs, median OS was 127 months in patients with BM and 170 months in patients without BM (log-rank p = 0.023).

**Figure 2:**
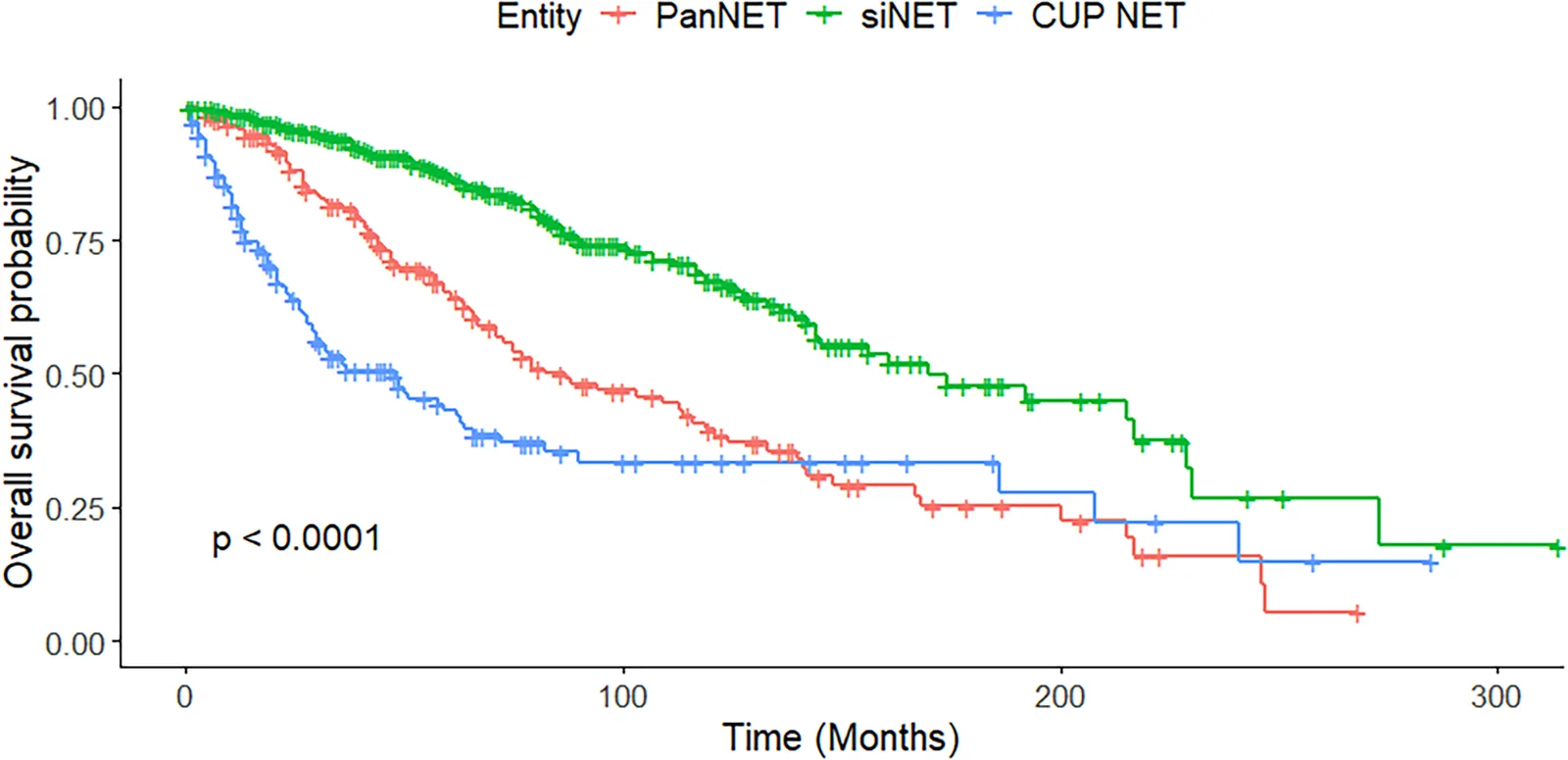
Kaplan-Meier estimates of overall survival by NET type in patients with bone metastases.

OS also differed according to tumor grade (log-rank p < 0.001; **Figure 3**). Median OS was 170 months (95% CI, 121–219) for G1, 113 months (95% CI, 90–136) for G2, and 31 months (95% CI, 10–52) for G3 NETs. No statistically significant difference in OS was observed according to functioning status (log-rank p = 0.30).

**Figure 3:**
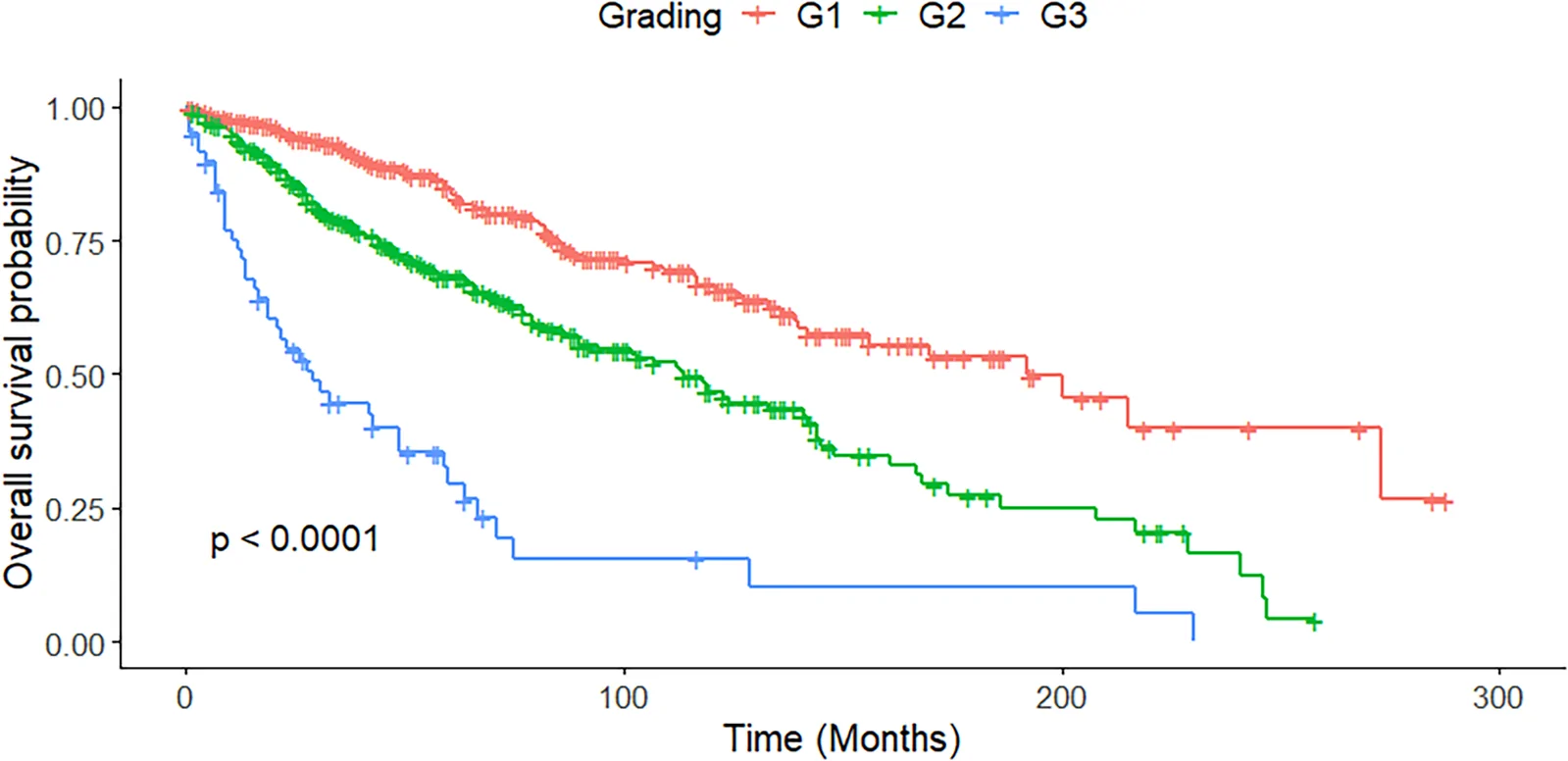
Overall survival according to tumor grading in patients with metastatic neuroendocrine tumors.

### Cox regression analyses

In entity-specific univariable Cox models, BM were associated with OS in PanNETs (HR, 1.66; 95% CI, 1.10–2.53; p = 0.017), siNETs (HR, 1.57; 95% CI, 1.04–2.35; p = 0.030), and CUP NETs (HR, 1.63; 95% CI, 1.07–2.48; p = 0.022).

In the entire cohort, the unadjusted HR for the association between BM and OS was 1.72 (95% CI, 1.36–2.18; p < 0.001). In the multivariable model including BM, age, sex, tumor grade, functioning status, and tumor entity, BM remained associated with shorter OS (HR, 1.45; 95% CI, 1.13–1.85; p = 0.0034). The complete model, including estimates for all covariates and the number of observations and events, is presented in **Table 2** and **Figure 4**.

**Table 2:** Cox proportional hazards regression analyses for overall survival, n = 634 patients, 270 events (8 patients excluded due to missing tumor grade).

Variable	Univariable HR (95% CI)	p value	Multivariable HR (95% CI)	p value
Bone metastases, present vs absent	1.72 (1.36–2.18)	<0.001	1.45 (1.13–1.85)	0.0034
Age, per 10-year increase	1.44 (1.30–1.59)	<0.001	1.42 (1.28–1.57)	<0.001
Male vs female sex	1.01 (0.79–1.28)	0.95	0.99 (0.78–1.27)	0.94
Grade G2 vs G1	1.73 (1.32–2.27)	<0.001	1.48 (1.12–1.96)	0.0066
Grade G3 vs G1	4.90 (3.37–7.11)	<0.001	4.22 (2.87–6.22)	<0.001
Functioning vs nonfunctioning	1.04 (0.82–1.32)	0.75	1.26 (0.96–1.66)	0.090
PanNET vs CUP NET	0.64 (0.48–0.86)	0.0032	0.80 (0.58–1.12)	0.19
siNET vs CUP NET	0.34 (0.26–0.46)	<0.001	0.42 (0.31–0.57)	<0.001

HR, hazard ratio; PanNET, pancreatic NET, CUP-NET, neuroendocrine tumor of unknown primary; siNET, small-intestinal NET.

**Figure 4:**
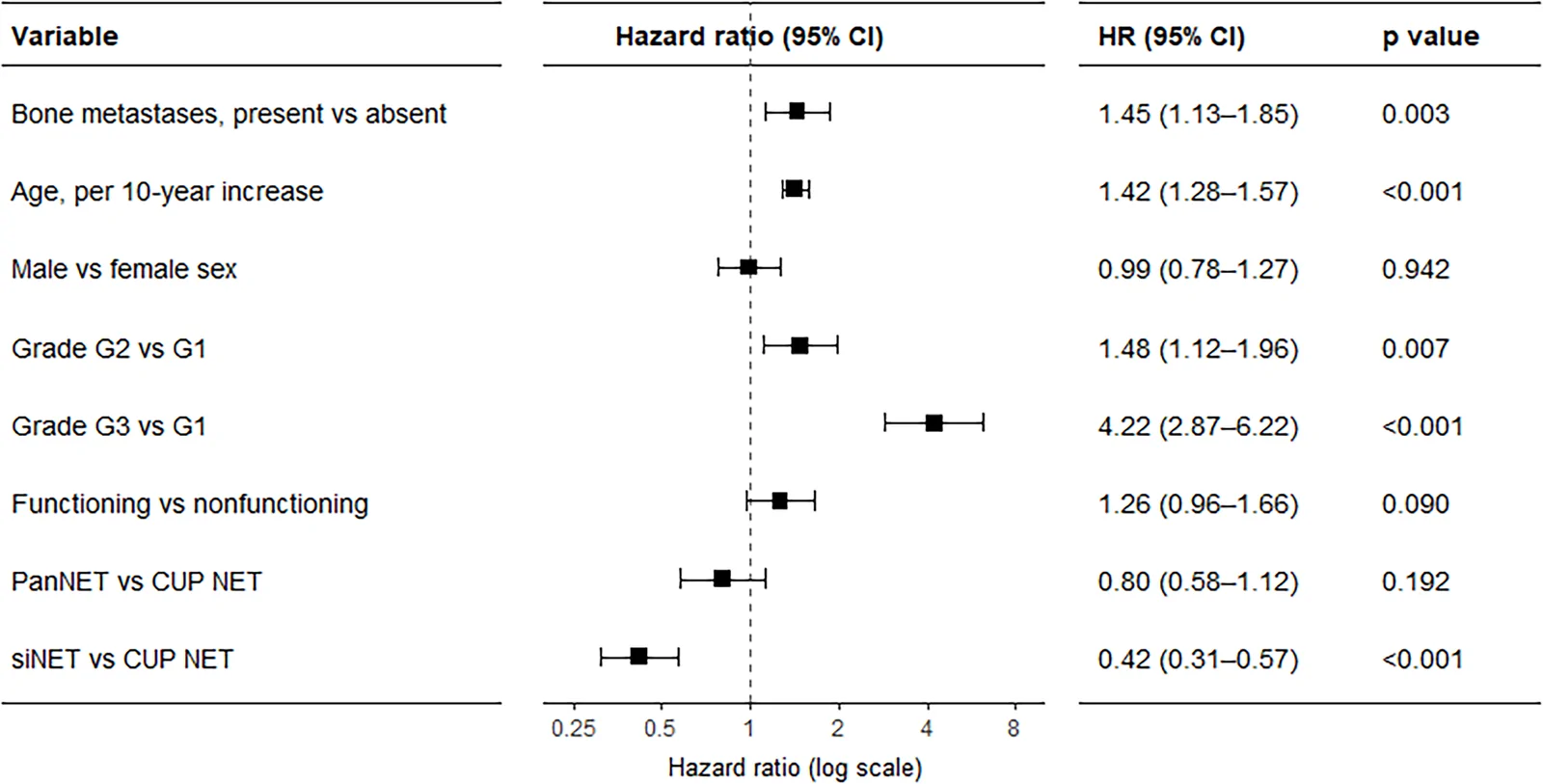
Forest plot of the multivariable Cox proportional hazards model for overall survival (n = 634, 270 events).

The proportional hazards assumption was not met for tumor entity. An additional model was therefore stratified by NET entity. In this model, BM remained associated with OS after adjustment for the covariates (HR, 1.43; 95% CI, 1.09–1.89; p = 0.011).

Within the CUP NET subgroup, the adjusted HR associated with BM was 1.83 (95% CI, 1.17–2.85; p = 0.008) after adjustment for age, sex, tumor grade, and functioning status. This model included 166 patients, and 89 deaths.

### Bone metastasis characteristics and skeletal-related events

Among patients with BM, lesion morphology differed across NET entities (**Table 3**). Osteoblastic lesions were documented in 23 of 62 PanNET patients (37%), 61 of 105 siNET patients (58%), and 26 of 71 CUP NET patients (37%). The remaining lesions had a mixed morphology or could not be evaluated morphologically due to isolated tracer accumulation. Entity-specific distributions of BM are shown in **Figure 5**.

**Table 3:** Bone metastases characteristics, SREs and antiresorptive therapy among BM cohort.

Entity	BM patients, n	Osteoblastic BM, n (%)	Patients with SRE, n (%)	Antiresorptive therapy, n (%)
PanNET	62	23 (37%)	11 (18%)	36 (58%)
siNET	105	61 (58%)	13 (12%)	29 (28%)
CUP NET	71	26 (37%)	34 (48%)	18 (25%)

BM, bone metastases; SRE, skeletal-related event; PanNET, pancreatic NET; siNET, small-intestinal NET, CUP-NET, neuroendocrine tumor of unknown primary.

**Figure 5:**
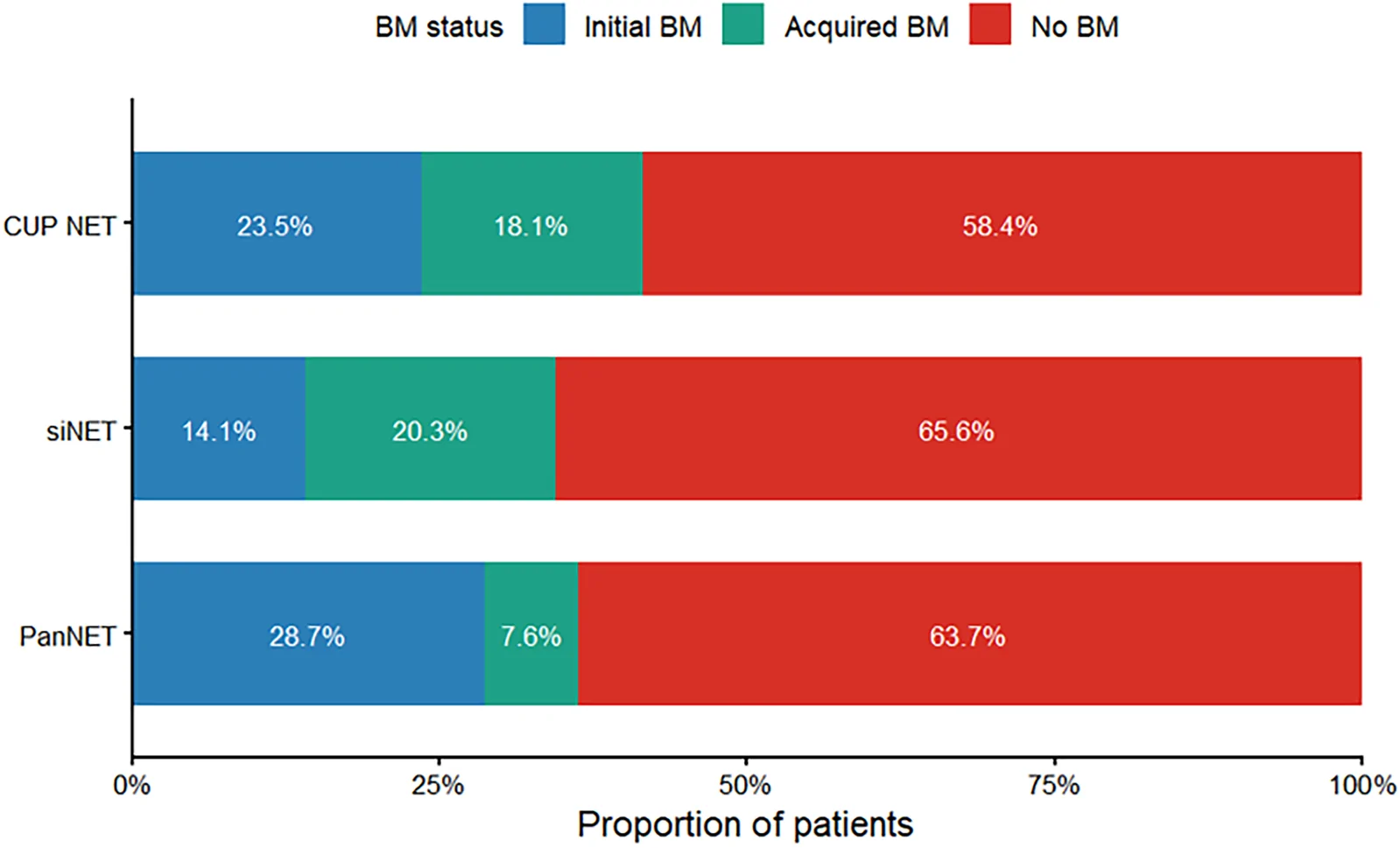
Timing of bone metastasis detection according to neuroendocrine tumor entity.

SREs were documented in 11 PanNET patients with BM (18%) and 13 siNET patients with BM (12%). Pain was the most common SRE, followed by spinal cord compression and pathological fractures. In the CUP NET cohort, SREs occurred in 48% (34 out of 71) of patients. Of these events, 56% involved pain, 19% involved spinal cord compression, 16% resulted in pathological fractures, and 9% required bone radiation. The morphology of the bone metastases was not correlated with the occurrence (p=0.314) or type (p=0.3; Fisher exact test) of SREs.

Antiresorptive therapy was administered to 36 patients with PanNETs and BM (58%), 29 with siNETs and BM (28%), and 18 with CUP NETs and BM (25%). Treatment consisted of bisphosphonates, or denosumab in cases of renal insufficiency. Because treatment allocation and timing were not randomized, descriptive treatment data were not interpreted as evidence of efficacy.

## Discussion

In this retrospective, single-center analysis of prospectively collected data from 642 patients with metastatic, well-differentiated NETs, BM were documented in 37.1% of patients. Their prevalence was numerically highest among patients with CUP-NETs, although differences between NET entities were not statistically significant. BM were associated with shorter OS both in the overall cohort and in entity-specific analyses, including the CUP-NET subgroup.

In multivariable analysis, BM remained associated with shorter OS after adjustment for age, sex, grade, functional tumor status, and NET entity (HR 1.45, 95% CI 1.13–1.85; p=0.0034). Increasing age and higher tumor grade were also associated with mortality, with the strongest association observed for G3 versus G1 tumors (HR 4.22, 95% CI 2.87–6.22; p<0.001). Compared with CUP-NETs, siNETs were associated with longer OS (HR 0.42, 95% CI 0.31–0.57; p<0.001), whereas the association for PanNETs was attenuated and no longer statistically significant after adjustment. Neither sex nor functional status was significantly associated with OS. These findings suggest that BM provide prognostic information beyond the clinicopathological variables included in the model, although residual confounding by unmeasured factors cannot be excluded.

A key contribution of this study is its focused analysis of CUP-NETs, a clinically understudied subgroup in which prognostic stratification is challenging because the primary tumor cannot be identified. Our findings indicate that BM status may represent a clinically relevant risk marker within this subgroup, which was characterized by a high prevalence of BM and poor survival outcomes (4, 6). The adverse prognostic association of BM persisted after adjustment for major clinicopathological variables and remained evident in the entity-stratified Cox model. This finding suggests that skeletal involvement may identify a clinically aggressive disease phenotype rather than merely reflecting differences in tumor site or grade. Nevertheless, the results should be interpreted as evidence of a prognostic association rather than causality. Patients with BM may have had a greater overall metastatic burden, poorer performance status, or different treatment exposure. Because these variables were not uniformly available in the retrospective dataset, residual confounding cannot be excluded.

The CUP-NET subgroup requires particular diagnostic consideration. A diagnosis of CUP-NET encompasses patients in whom the primary tumor remains unidentified despite clinical, radiological, endoscopic, and pathological evaluation. However, the extent of the diagnostic workup may vary depending on the clinical presentation and availability of diagnostic modalities. Occult small-intestinal primaries may remain undetected even after conventional imaging and 68Ga-DOTATOC PET/CT, and not all patients undergo capsule endoscopy, enterography, deep enteroscopy, or surgical exploration. Consequently, some patients categorized as having CUP-NET may have harbored an unrecognized small-intestinal or other primary tumor. The proportion of functioning tumors and the distribution of metastases among patients with CUP-NET could be compatible with an occult midgut origin in a subset of cases. However, the present study does not provide sufficient molecular evidence to establish a “midgut-like phenotype”. Even when supported by CDX2 expression or serotonin-related markers, such an interpretation should remain cautious, and CUP-NETs should not be equated with siNETs on the basis of clinical presentation alone.

Earlier consensus statements reported skeletal involvement in <15% of patients with NETs, a figure now widely considered an underestimate attributable to the limited sensitivity of conventional imaging modalities (6–8). With the routine use of SSTR-PET/CT, skeletal disease is increasingly recognized as a common manifestation of advanced NETs rather than a rare, late complication. Our previous studies of PanNETs and siNETs reported BM prevalence rates of approximately one-third in advanced disease and demonstrated an association between skeletal involvement and impaired survival (2, 3). In the present cohort, the direction and magnitude of the prognostic association appeared broadly consistent across NET entities, as indicated by comparable hazard ratios in the Cox analyses. BM prevalence was numerically highest among patients with CUP-NETs (42.8%), followed by those with PanNETs (36.3%) and siNETs (34.4%); however, these differences were not statistically significant. Within the CUP-NET subgroup, BM identified a high-risk population with significantly shorter OS than that of patients without skeletal involvement. This survival difference is particularly relevant in a clinically heterogeneous subgroup in which established prognostic markers are limited by the absence of an identifiable primary tumor.

Potential molecular mechanisms underlying skeletal dissemination in neuroendocrine neoplasms include epithelial–mesenchymal transition, CXCR4/CXCL12-mediated homing, and dysregulation of the receptor activator of nuclear factor-κB (RANK)/RANK ligand (RANKL)/osteoprotegerin axis (1, 6, 9). These pathways may facilitate tumor-cell homing, colonization, and persistence within the bone microenvironment and thus provide biologically plausible hypotheses derived from translational studies and models of other tumor types. However, these pathways were not investigated in the present cohort and should therefore be regarded as hypothesis-generating rather than as mechanisms demonstrated by our data.

Patients with CUP-NETs constitute a diagnostically and potentially biologically heterogeneous group within the NET spectrum. In broader populations of patients with cancer of unknown primary, extranodal metastatic patterns, including skeletal involvement, have been associated with inferior survival. Our findings extend this observation to well-differentiated NETs and suggest that BM may contribute to the poor outcomes observed among patients with CUP-NETs compared with those with site-defined NETs, despite broadly similar grade distributions. However, this interpretation requires validation in cohorts with more comprehensive adjustment for disease burden, performance status, and treatment exposure.

The timing of skeletal involvement may provide additional insight into tumor behavior. Previous studies have suggested that synchronous BM are associated with greater proliferative activity and tumor burden and with poorer survival than metachronous BM. Consistent with these observations, patients with CUP-NETs in our cohort appeared more likely to develop BM early in the disease course. This finding may indicate that skeletal dissemination in CUP-NETs is associated with aggressive tumor behavior rather than solely representing the late accumulation of metastatic disease (10). Nevertheless, analyses of BM detected during follow-up require appropriate time-dependent methods, and the timing-related findings should therefore be interpreted cautiously.

Nearly half of the patients with BM-positive CUP-NETs experienced skeletal-related events, and bone pain was the most commonly documented clinical manifestation. Antiresorptive therapy was administered to only a subset of patients and was often initiated after a delay following BM diagnosis. Prospective studies are needed to determine which patients with NETs and skeletal involvement benefit from bone-targeted therapy and to establish the optimal timing of such interventions.

From a clinical perspective, our findings support careful assessment of the skeleton during staging and follow-up, particularly in patients with symptoms or other findings suggestive of osseous disease. The strengths of this study include the continuous prospective collection of data from a relatively large cohort, the dedicated analysis of patients with CUP-NETs, multidisciplinary case review, long-term survival data, and standardized longitudinal imaging, with all patients undergoing serial 68Ga-DOTATOC PET/CT examinations combined with diagnostic contrast-enhanced CT.

Several limitations should be acknowledged. First, although the data were collected in a prospectively maintained database, the present analysis was retrospective and conducted at a single tertiary referral center. Consequently, selection bias cannot be excluded, and the generalizability of the findings to other clinical settings may be limited. Second, potentially relevant confounders, including hepatic and total metastatic burden and treatment exposure, were not fully accounted for in the primary model, leaving the possibility of residual confounding. Third, despite standardized longitudinal imaging, BM diagnosed after cohort entry constitute a time-varying exposure. Analyses treating BM status as a fixed variable may therefore be susceptible to time-dependent bias, and future studies should model BM occurrence as a time-varying covariate.

Overall, BM were common and associated with shorter OS in this cohort of patients with metastatic, well-differentiated NETs. This association was also observed within the CUP-NET subgroup. However, whether BM provide prognostic information independently of total disease burden and treatment exposure requires further investigation.

## Conclusion

Bone metastases were common among patients with metastatic, well-differentiated NETs and were associated with shorter OS across the examined NET entities, including CUP-NETs. These findings suggest that BM status may provide prognostic information beyond routinely available clinicopathological characteristics. However, the retrospective design precludes causal interpretation and does not support the immediate incorporation of BM status into formal risk-stratification systems. Prospective studies using time-dependent analyses and comprehensive adjustment for confounders are warranted.

## Data Availability

The raw data supporting the conclusions of this article will be made available by the authors, without undue reservation.
